# Maternal caloric restriction partially rescues the deleterious effects of advanced maternal age on offspring

**DOI:** 10.1111/acel.12217

**Published:** 2014-03-24

**Authors:** Kristin E Gribble, George Jarvis, Martha Bock, David B Mark Welch

**Affiliations:** 1 Josephine Bay Paul Center for Comparative Molecular Biology and Evolution, Marine Biological LaboratoryWoods Hole, MA, 02543, USA; 2 Northeastern University360 Huntington Ave., Boston, MA, 02115, USA

**Keywords:** aging, caloric restriction, maternal effect, monogonont rotifer

## Abstract

While many studies have focused on the detrimental effects of advanced maternal age and harmful prenatal environments on progeny, little is known about the role of beneficial non-Mendelian maternal inheritance on aging. Here, we report the effects of maternal age and maternal caloric restriction (CR) on the life span and health span of offspring for a clonal culture of the monogonont rotifer *Brachionus manjavacas*. Mothers on regimens of chronic CR (CCR) or intermittent fasting (IF) had increased life span compared with mothers fed *ad libitum* (AL). With increasing maternal age, life span and fecundity of female offspring of AL-fed mothers decreased significantly and life span of male offspring was unchanged, whereas body size of both male and female offspring increased. Maternal CR partially rescued these effects, increasing the mean life span of AL-fed female offspring but not male offspring and increasing the fecundity of AL-fed female offspring compared with offspring of mothers of the same age. Both maternal CR regimens decreased male offspring body size, but only maternal IF decreased body size of female offspring, whereas maternal CCR caused a slight increase. Understanding the genetic and biochemical basis of these different maternal effects on aging may guide effective interventions to improve health span and life span.

## Introduction

Interventions to improve health span and increase longevity rely on plasticity in age-related traits and life span. Such plasticity may evolve as an adaptive response to environmental heterogeneity or may be an indirect outcome of changing resources and stressors. Extended life span under caloric restriction (CR), for example, is thought to have evolved to delay reproductive senescence during times of resource limitation to allow reproduction to resume later in life when food is restored (Fisher, 1958; Kirkwood, 1977; Holliday, 1989; Stearns, 1989). Alternatively, CR may cause a hormetic response, resulting in upregulation of protective mechanisms to defend against the stress of limited food, secondarily resulting in increased longevity (Masoro, 2007).

Phenotypic plasticity may occur not only in an individual, but also across generations. Transgenerational phenotypic plasticity, particularly the influence of the maternal environment on the phenotype of offspring, known as maternal effects, may be detrimental or beneficial to progeny fitness. A maternal effect known to impact offspring fitness is the age at which mothers give birth. In many plant and animal species, older mothers have shorter-lived offspring, a phenomenon called the ‘Lansing effect’ (Lansing, 1947; Priest *et al*., 2002).

Aging biology and medical research tend to focus on the negative maternal effects of harmful maternal environments and behavior, including smoking, drinking, obesity and advanced maternal age. Less attention has been paid to the role that adaptive transgenerational phenotypic plasticity may play in aging (Brakefield *et al*., 2005), although there is a large body of ecological literature on increasing progeny fitness through maternal effects. Examples include *Drosophila*, where females that mate more frequently produce daughters with higher fecundity (Priest *et al*., 2007); two different aquatic invertebrates, *Daphnia* and rotifers, in which mothers exposed to predators produce offspring with defensive horns and spines (Gilbert, 1967; Krueger & Dodson, 1981; Stemberger & Gilbert, 1987); and plants, where progeny have higher fitness when grown in the same light environment as the maternal plant (Galloway & Etterson, 2007).

To advance our understanding of the role of adaptive transgenerational phenotypic plasticity in aging, we examined the effects of maternal CR on the size, life span and fecundity of offspring in the monogonont rotifer *Brachionus manjavacas*, a member of the well-studied *Brachionus plicatilis* species complex. Monogonont rotifers are aquatic microinvertebrates that have been used in aging research for nearly 100 years (Austad, 2009). *Brachionus manjavacas* is cyclically parthenogenetic, generally reproducing asexually, with amictic (asexual) diploid females producing diploid amictic female eggs through mitosis. In response to environmental conditions including crowding, some amictic individuals produce mictic (sexual) females. These mictic females, if unfertilized, produce haploid male offspring via meiosis. If fertilized by males, mictic females lay diploid resting eggs that overwinter in the sediments and hatch into amictic females, restoring the asexual cycle. All females are produced asexually from amictic mothers or are hatched from resting eggs; males are only produced by mictic mothers.

Monogonont rotifers are a tractable and relevant model system for studying the biology of aging, as their responses to interventions, genetic mechanisms and biochemical pathways are largely conserved with mammals (Austad, 2009). In addition, rotifers have not undergone the genome reduction seen in some other invertebrates and thus may share more genes in common with humans than some model systems (Kortschak *et al*., 2003). As monogonont rotifers do not care for their eggs or offspring, they provide a good model for separating the influences of parental care from maternal effects on offspring fitness. Under our culture conditions and depending on feeding regimen, amictic *B*. *manjavacas* females hatched from amictic eggs have a prereproductive (neonate) stage of 1–2 days, reach maximum reproductive output at day 4–6, produce 22–32 eggs and live an average of 8–12 days (Gribble & Mark Welch, 2013). Because we maintain our cultures as clonal lineages, intraspecific variability in life span and fecundity can be attributed to environmental influences and maternal effects.

Our goal in this study was to determine whether the negative effects of increasing maternal age on offspring fitness could be countered by maternal caloric restriction and whether observed maternal effects varied by the type of caloric restriction or the sex of offspring. Female offspring of the closely related rotifer *B. plicatilis* have increased life span when their mothers are subjected to intermittent fasting (IF) (Kaneko *et al*., 2011), but differences between sexes are often found in the response to interventions to aging in other species. We subjected mothers to both chronic caloric restriction (CCR) and to IF, as different CR regimens likely work through different mechanisms and may have different outcomes in the same isolate (Anson *et al*., 2003, 2005; Greer & Brunet, 2009; Cleary & Grossmann, 2011; Gribble & Mark Welch, 2013; Gribble *et al*. 2014).

## Results

### Life span

**Figure 1:**
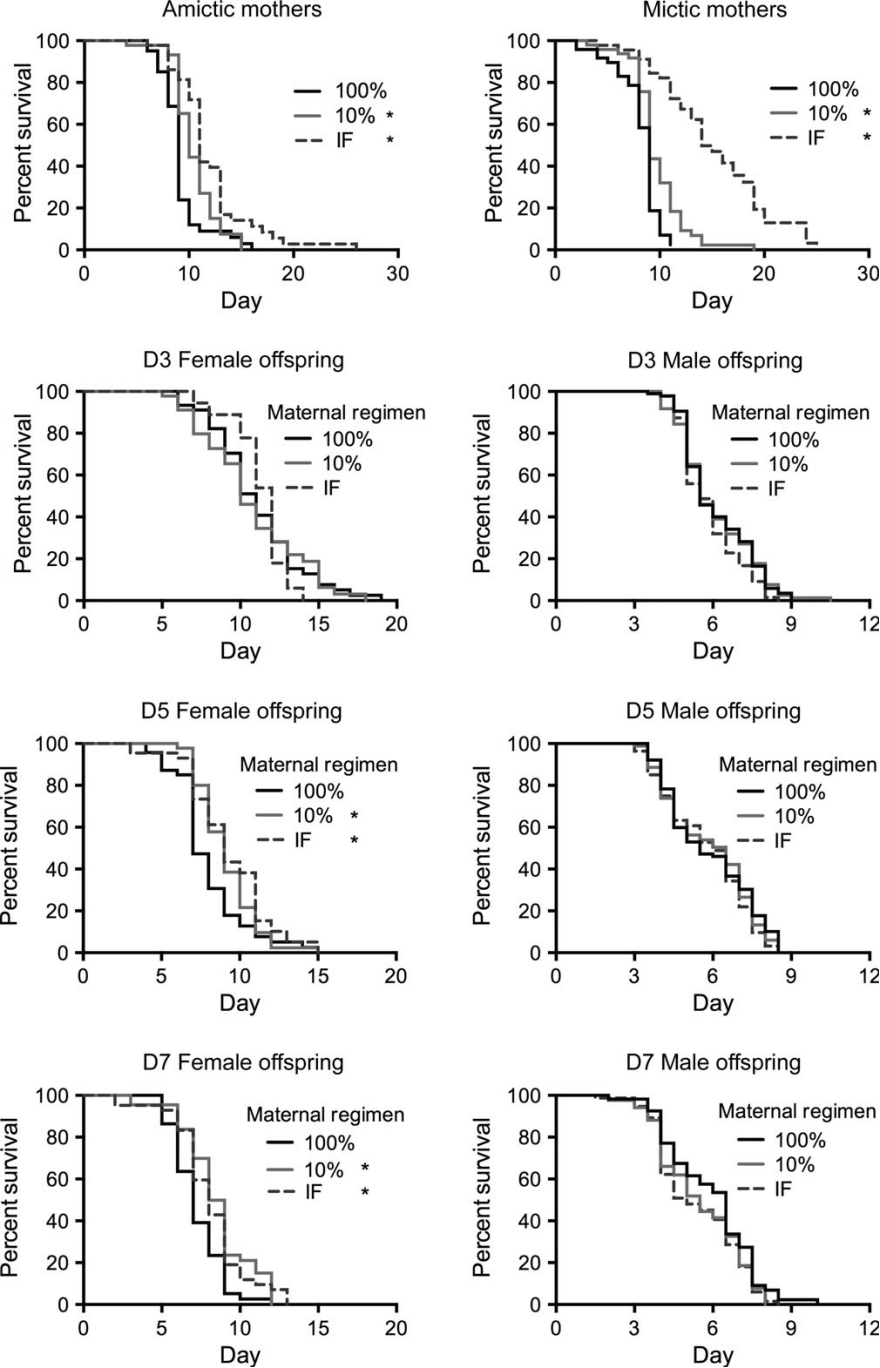
Kaplan–Meier survival curves for *Brachionus manjavacas* mothers and their offspring. Top, amictic mothers (left) and mictic mothers (right) fed at 100% or 10% of *ad libitum* levels or under intermittent fasting (IF). Lower curves, female (left) and male (right) offspring of 3-day-, 5-day-, or 7-day-old mothers under the indicated CR regimen. * indicates significant difference between CR treatment and 100% *ad libitum* feeding of maternal females (*P* < 0.05, Mantel–Cox test).

**Figure 2:**
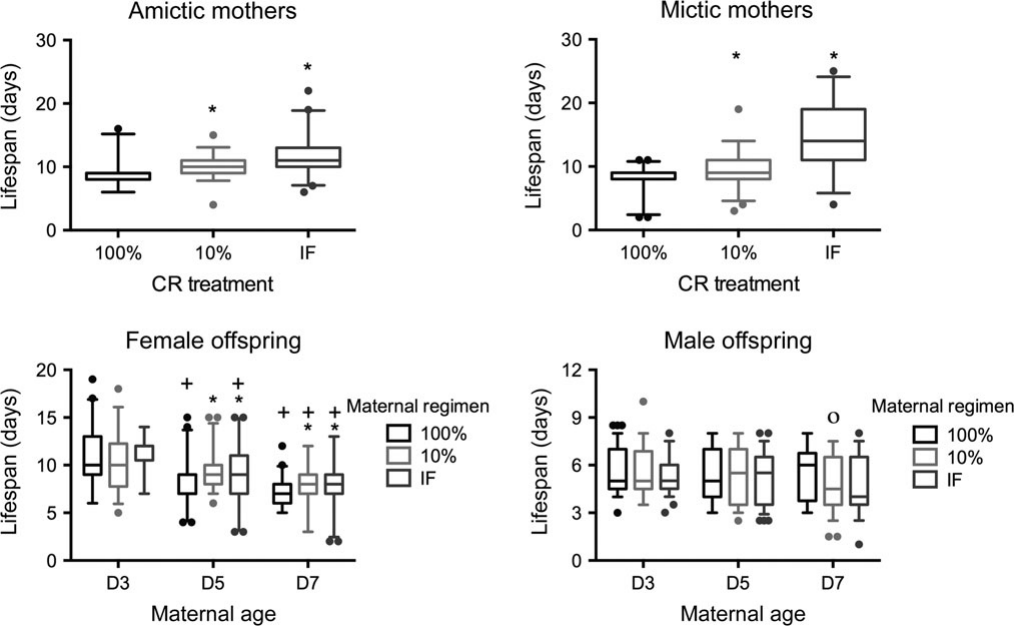
Mean life span of *Brachionus manjavacas* mothers and their offspring. Top, amictic mothers (left) and mictic mothers (right) fed at 100% or 10% of *ad libitum* levels or under intermittent fasting (IF). Bottom, female (left) and male (right) offspring of 3-day-, 5-day-, or 7-day-old mothers under the indicated CR regimen. Female offspring were all fed at 100% food levels; male offspring do not eat. Horizontal lines show mean life span, boxes indicate 25th and 75th percentiles, whiskers show 5th and 95th percentiles, and dots show outliers. * denotes the mean was significantly different than the 100% treatment for the same cohort (Kruskal–Wallis test, *P* < 0.05). + indicates the mean was significantly different than day 3 for the same food conditions, and ο indicates the mean was significantly different than that on day 5 for the same food conditions (Kruskal–Wallis with Dunn’s multiple comparison’s test, *P* < 0.05).

We subjected mothers to either chronic caloric restriction (CCR) at 10% of *ad libitum* (AL) food levels (a 90% reduction in food) or to IF by feeding AL and starving on alternate days using standard protocols (see Experimental procedures for specifics). All offspring fed at AL food levels. For both amictic and mictic mothers, 10% CCR and IF significantly changed the shape of the survival curves (Fig. 1), and mean life span was significantly increased (Fig. 2), consistent with previous results (Gribble & Mark Welch, 2013). The largest change in mean life span was for mictic IF mothers, from 8.0 days under AL conditions to 14.6 days under IF an increase of 83%. Maximum life span (95th percentile) increased under CR, except for amictic mothers under 10% CCR [Fig. 2, Table S1 (Supporting information)].

The mean and maximum life span of female offspring decreased with increasing maternal age, with older (day 5, day 7) mothers producing significantly shorter-lived offspring than younger (day 3) mothers (Figs 1 and 2). Maternal CR significantly increased the mean life span of female offspring of older mothers, but not of younger mothers. We found no correlation between life spans of individual mothers and the life spans of their female offspring (data not shown). After day 3, both maternal CCR and maternal IF increased the maximum life span (95th percentile survivorship) of offspring. Neither maternal age nor maternal CR influenced the mean or maximum life span of male offspring.

### Fecundity

**Figure 3:**
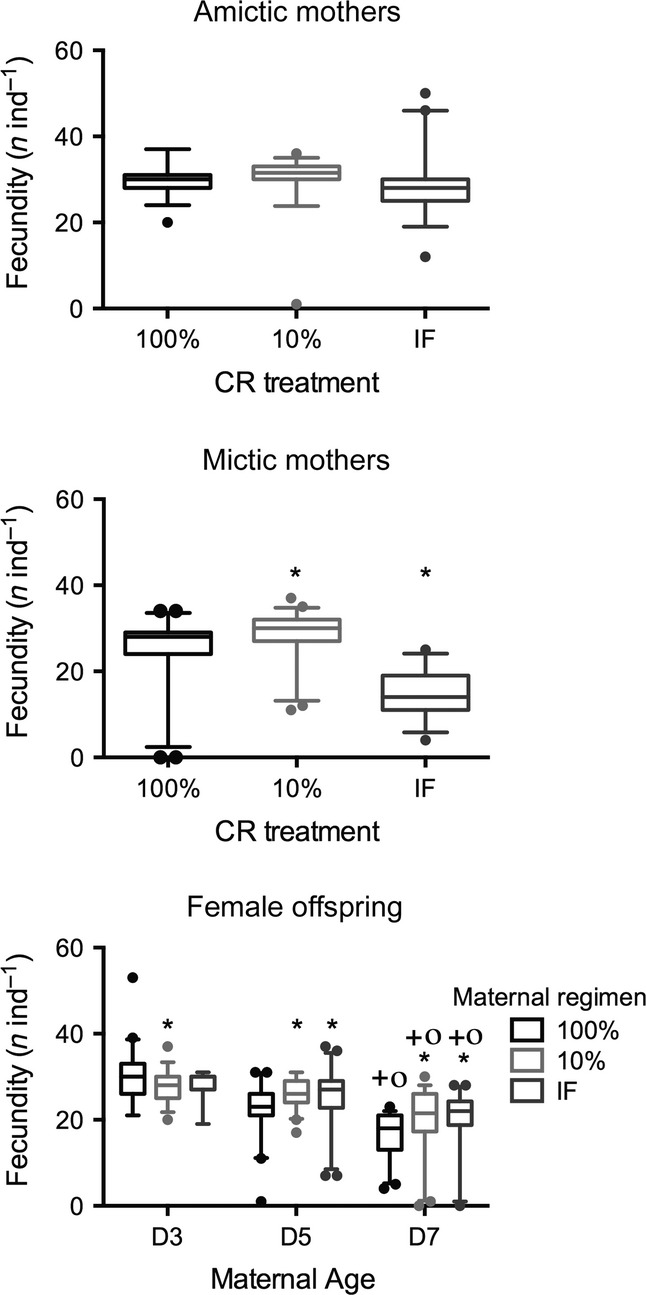
Mean fecundity of *Brachionus manjavacas* mothers and their offspring. Top and middle, amictic mothers and mictic mothers, respectively, fed at 100% or 10% of *ad libitum* levels or under intermittent fasting (IF). Bottom, female offspring of 3-day-, 5-day-, or 7-day-old mothers under the indicated CR regimen. Horizontal lines show mean fecundity, boxes indicate 25th and 75th percentiles, whiskers show 5th and 95th percentiles, and dots show outliers. * denotes the mean was significantly different than the 100% treatment for the same cohort (Kruskal–Wallis test, *P* < 0.05). + indicates the mean was significantly different than day 3 for the same food conditions, and ο indicates the mean was significantly different than that on day 5 for the same food conditions (Kruskal–Wallis with Dunn’s multiple comparison’s test, *P* < 0.05).

**Figure 4:**
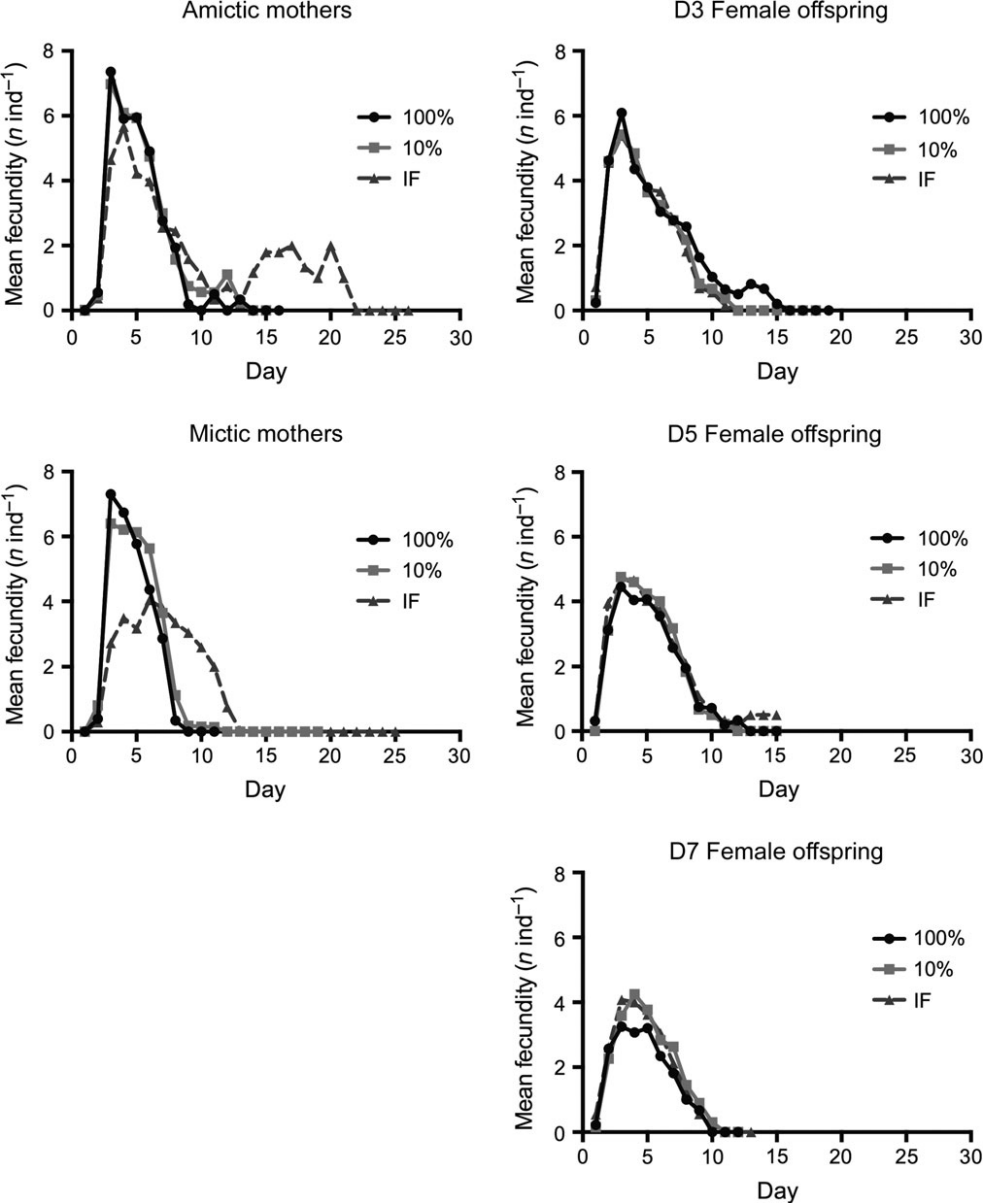
Mean daily reproduction of *Brachionus manjavacas* mothers and their offspring. Left, amictic mothers (top) and mictic mothers (bottom) fed at 100% or 10% of *ad libitum* (AL) levels or under intermittent fasting (IF). Right, female offspring of 3-day-, 5-day-, or 7-day-old mothers under the indicated CR regimen. All offspring were fed at 100% AL.

Mean total fecundity of mictic mothers increased significantly under 10% CCR and decreased significantly under IF (Fig. 3); this trend also occurred in amictic mothers but was not significant, in part because of an extended reproductive stage in a small number of IF mothers (Fig. 4). Maternal CR of either regimen increased fecundity of daughters of older mothers by increasing mean daily reproduction during the time of maximum reproduction (Fig. 4). Fecundity of daughters of young mothers was slightly decreased by maternal CR (Fig. 3). For daughters born to older mothers, the time of maximum reproduction shifted from day 3 to day 4, independent of maternal CR. We did not examine the impact of maternal CR on male fertility.

### Body size

**Figure 5:**
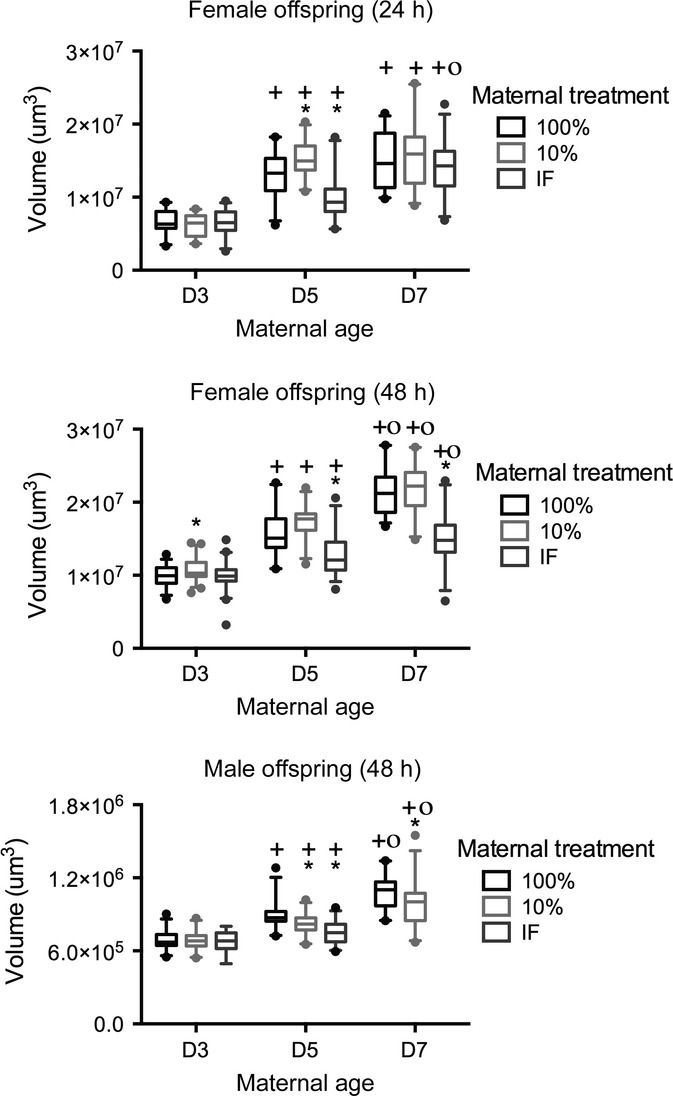
Body size of *Brachionus manjavacas* offspring. Top, Amictic female offspring at 24 h; middle, amictic female offspring at 48 h; and bottom, male offspring at 24 h, from mothers fed at 100% or 10% of *ad libitum* levels or under intermittent fasting (IF). Horizontal lines show mean life span, boxes indicate 25th and 75th percentiles, whiskers show 5th and 95th percentiles, and dots show outliers. * denotes the mean was significantly different than the 100% treatment for the same day cohort (Kruskal–Wallis test, *P* > 0.05). + indicates the mean was significantly different than day 3 for the same food conditions, and ο indicates the mean was significantly different than that on day 5 for the same food conditions (Kruskal–Wallis with Dunn’s multiple comparison’s test, *P* < 0.05).

To determine whether a trade-off between increased life span and body size under decreased energy intake might be transmitted to progeny, we measured the body size of offspring. Female body size in *B. manjavacas* increases rapidly in the first 2 days and continues to increase slightly throughout life; we measured volume at 24 h to represent neonate size and at 48 h to represent size at the time of first reproduction. At both timepoints, daughters of older mothers were significantly larger than daughters of younger mothers, regardless of maternal diet (Fig. 5). By 48 h, daughters of 7-day-old AL mothers were nearly twice the volume of daughters born to 3-day-old AL mothers and were significantly larger than daughters born to 5-day-old AL mothers. Maternal IF, but not 10% CCR, reduced this difference, with daughters of older IF mothers significantly smaller than daughters of older AL or CCR mothers.

Males undergo little growth during their short lives. The body size of males at 24 h increased with maternal age, with 7-day-old AL mothers producing offspring ~60% larger than 3-day-old mothers and significantly larger than offspring of 5-day-old mothers. Maternal CR of either regimen significantly decreased the size of males born to older mothers.

## Discussion

In this study, we investigated the consequences of maternal age and maternal CR on offspring life span and fecundity in the rotifer *B. manjavacas*. We found that maternal age impacted the life span, fecundity and size of offspring and that maternal CR could reduce the severity of these effects to varying degrees, depending on the type of CR and gender of the offspring. Different maternal CR regimens elicited different effects on both mothers and their offspring.

### Effect of maternal age on female offspring life span and fecundity

We found that life span and fecundity in female offspring declined with increasing maternal age in *B. manjavacas*. Similar maternal age effects have been seen across a wide range of species (Priest *et al*., 2002). Lansing showed that this declining fitness was cumulative across generations of rotifers to the point that cultures continually derived from offspring of older mothers died out after several generations, whereas cultures from young mothers could be maintained indefinitely (Lansing, 1947, 1954). An increase in the accumulation of carbonylated proteins in the offspring of older mothers, suggesting oxidative damage, has been seen in the copepod *Acartia tonsa*, another aquatic microinvertebrate (Rodríguez-Graña *et al*., 2010). With increasing maternal age, protein banding patterns in bdelloid rotifers show decreased protein concentration and increased variability in both maternal females and their offspring (Ricci *et al*., 1999), suggesting protein disregulation that is passed on to daughters of older mothers. The negative life span effects on offspring of increasing maternal age may be due to a combination of processes acting on the germline: epigenetic changes affecting gene expression, accrual of protein damage, mutation accumulation (Medawar, 1952) and antagonistic pleiotropy (Williams, 1957). While these mechanisms are generally considered for their effects directly on aging in the individual, this study and others suggest that they should be considered in a transgenerational context and that maternal effects should be incorporated into evolutionary theories of aging (Priest *et al*., 2002).

In our study, mean life span of offspring born to young mothers was greater than the mean life span of their mothers, recapitulating the ‘rescue effect’ seen by Lansing (1947), in which the life span of an old orthoclone could be increased by collecting early-born instead of late-born offspring. The maternal rotifers we used in our experiments were themselves the offspring of females of mixed ages, thus their mean life span was influenced by a wide range of maternal ages. The mean life span in early-born (day 3) offspring was 19% higher than that of their AL-fed mothers, even without the added benefits of maternal CR. Lansing (1947, 1954) hypothesized that the cumulative aging effect across generations was caused by a factor that accumulates during an individual’s life and is transmitted to the next generation, an idea compatible with both mutation accumulation and antagonistic pleiotropy theories. How the aging clock is reset in offspring born to young mothers remains a mystery.

The shape of the survivorship curves, with high initial survivorship and then relatively rapid mortality, is common for *Brachionus* rotifers whether or not the rotifers are handled daily (e.g., Serra *et al*., 1994; Snare *et al*., 2013) and is a function of high survivorship during the reproductive period and a short overall life span.

### Impacts of maternal CR on female offspring

We found significant differences in life span and fecundity of female offspring depending on maternal CR regimen, even though all offspring fed AL throughout their lives. While IF increased life span more than did 10% CCR in mothers, daughters of mothers from either CR treatment had about a 17% increase in life span. This transgenerational life span increase is of similar magnitude to that seen in a strain of *B. plicatilis* fed a different species of algae under IF conditions (Kaneko *et al*., 2011). Our demonstration that maternal CCR also increases offspring life span is significant because the beneficial effects of CCR and IF may act through overlapping, but not identical pathways in rotifers, as is the case in other animals (Greer *et al*., 2007; Greer & Brunet, 2009; Cleary & Grossmann, 2011; Dogan *et al*., 2011; Gribble & Mark Welch, 2013; Gribble *et al*. 2014). We also found that daughters of both 10% CCR and IF mothers had increased fecundity relative to the daughters of AL mothers, even though the mothers themselves had slightly lower mean fecundity under IF than under 10% CCR.

The beneficial effects of maternal CR were not immediate: life span was not significantly extended in daughters of 3-day-old mothers. As all mothers fed AL for the first 24 h, CR mothers were only exposed to CCR for 48 h or to one round of 24-h fasting by day 3. This suggests that the physiological reaction triggering the epigenetic response is not an instantaneous result of exposure to low food levels. Whether working through nutrient sensing, metabolic and/or stress pathways, or other mechanisms, the initiation of the transgenerational response appears to require extended CR. Alternatively, there may be a developmental window during which exposure to maternal CR can lead to changes in offspring aging. Perhaps the day 3 offspring, already developing embryos within the first 24 h of the experiment, had exited this window before CR began. Future studies might examine how short-term CR or starvation exposures at different maternal ages translate into effects on offspring aging, to determine the timing and trigger for the maternal effect.

Maternal CR partially rescues daughters of older mothers from the detrimental effects of increasing maternal age. Daughters of older AL mothers had a 30% reduction in life span compared with daughters born to younger mothers, but daughters of both older CCR and IF mothers had only a 10% reduction. Whether maternal CR directly counters the effects of increasing maternal age is not known. CR decreases oxidative damage, confers increased resistance to oxidative stress and lessens the incidence of age-related diseases like diabetes and cancer (Anson *et al*., 2003; Rodríguez-Graña *et al*., 2010; Cleary & Grossmann, 2011; Dogan *et al*., 2011; Kailasam *et al*., 2011). Determining whether the effects of maternal age and maternal CR act in opposition through the same pathways or epigenetic mechanisms will provide insights to the diversity of aging regulation processes and possibly allow targets for maternal therapies to improve offspring health and aging.

### Impacts of maternal age and maternal CR differ for male and female offspring

In contrast to female offspring, male offspring of mictic females were not negatively impacted by maternal age, nor did they inherit life span-extending benefits from maternal CR. Gender differences in aging, the response to prenatal environment and the response to interventions to aging are widely reported, though the reasons for such differences are not fully understood. Epistatic interactions of an unknown nature lead to differences in the expression and effect of the same gene(s) in males and females. These differences in phenotype and mechanism, and their resulting effects on fitness, will need to be incorporated into working theories on the evolution of aging.

Given that male rotifers are not subject to either the benefits of maternal CR or the negative effects of increasing maternal age, we might speculate that these two maternal effects act through overlapping pathways that are not active in male rotifers. Male rotifers do not eat and thus presumably lack expression of metabolic genes involved in food digestion and metabolism; the maternal CR effect may be working, at least in part, through these pathways in daughters. Thus, if the maternal CR effect acts on IGF-1 and/or AMPK signaling in females, we might see that these mechanisms are essentially shut down in male offspring. Alternatively, the maternal CR effect may be indirect, acting through stress-response (hormesis) mechanisms or invoking epigenetic changes that are passed on to daughters, but not sons. Additionally, the meiotic origin of males may somehow block the maternal effects expressed in mitotically derived female offspring. Whether the maternal age effect works through the same, overlapping or separate pathways remains to be determined through comparative transcriptomic, protein activity and gene knock-down studies.

Maternal age and CR did not affect male life span, but did influence other aspects of fitness. The body size of both male and female offspring increased significantly with increasing maternal age. Partridge & Fowler (1992) also found larger body size in lines of *Drosophila* derived from old mothers than from those derived from young mothers. We expected that the decreased energy intake of CR mothers would result in decreased body size of offspring compared with those of AL mothers. In a classic ‘trade-off’ situation, CR mothers, with their extended life span, would have fewer energy resources to invest in offspring. However, we observed that while maternal CR of either regimen reduced the size of males born to older mothers, only maternal IF reduced the size of daughters. The difference in body size outcome depending on maternal CR regimen suggests differences in the mechanism of life span extension between CCR and IF. How these changes in body size might translate to fitness effects in the natural environment is likely quite complicated. For example, as body size increases, swimming speed also increases, which could aid in escape from larger predators. However, increased swimming speed leads to more encounters with predatory zooplankton and thus might increase predation risk (Preston *et al*., 1999). Understanding how differences in body size between male and female offspring due to maternal age and CR effects affect swimming speed and mating would provide insights into its adaptive significance.

### Evolution and adaptive significance of transgenerational plasticity in life span and reproduction

We hypothesize that the increase in life span and fecundity of female offspring due to maternal CR is adaptive, endowing offspring with increased fitness during times of limited food resources. Female offspring are likely to hatch into the same conditions that their mothers experienced, given rotifers’ short generation time of around 48 h. While maternal CR leads to longer life span in daughters, it is unclear that this is necessary for increased fitness of offspring, as *B. manjavacas* responds directly to CR (Gribble & Mark Welch, 2013; Gribble *et al*. 2014). The closely related species *B. plicatilis* shows a cumulative life span extension when both the mother and the daughter are subjected to CR, suggesting both maternal transmission and a direct effect of food conditions on the daughter (Kaneko *et al*., 2011).

Monogonont rotifers are known to have other adaptive maternal effects prompted by environmental conditions. Female rotifers exposed to a protein cue (kairomone) from predatory zooplankton produce offspring with large spines that are effective defenses against predation (Gilbert, 1967, 1999). Different, specific spine morphologies grow in response to different predator species, with each phenotype effective in defending against that particular predator species, but not others (Stemberger & Gilbert, 1987). In the case of inducible defenses, the mother does not herself produce mechanical defenses against predation; only her offspring are protected. As offspring are likely to hatch into the same predatory environment inhabited by their mothers, this transgenerational morphological plasticity increases fitness of offspring.

The disposable soma and life history theories predict that longer life span cannot be achieved under resource limitation without a corresponding decline in another energy-consuming aspect of fitness such as reproduction, body size or swimming speed (Kirkwood, 1977; Shanley & Kirkwood, 2000). In this experiment, the CR regimen determined the type and magnitude of resource allocation trade-off, both in the restricted generation and in their offspring. While IF in mothers, both amictic and mictic, slightly suppressed daily reproduction and extended reproductive life span, the fecundity of daughters was significantly increased by maternal CR, under both CCR and IF, and the reproductive period was unchanged from that of offspring of AL-fed mothers. Female offspring body size at the time of first reproduction was slightly increased with maternal CCR, but significantly decreased under maternal IF. Thus, the only trade-off apparent was a decrease in body size under maternal IF; we do not have direct evidence from this study that such a decrease negatively impacts fitness.

Maternal age and environment clearly influence progeny health, though finding cases of epigenetic adaptive transgenerational phenotypic plasticity due to CR or other interventions in additional species will be complicated by the genomic diversity caused by sexual reproduction. While evolutionary arguments may be made to support adaptive epigenetic effects of food restriction in humans, no consistent pattern in adult aging has been found in studies of the impact of prenatal exposure to CR during periods of human famine (Brakefield *et al*., 2005), and other maternal effects that increase human life span and health span have yet to be identified.

### Conclusions and implications

While an individuals’ genome affects its aging phenotype and longevity, there is a growing realization that epigenetic processes may contribute greatly to aging and life span. Such epigenetic processes may be controlled by environmental factors and act directly upon an individual, or as this study has demonstrated, may be controlled by parental environment. Understanding the mechanisms of transgenerational phenotypic plasticity affecting health and life span, the conservation of these effects among taxa and the diversity of outcomes between genders are critical to understanding the origins of aging and to developing treatments for age-related diseases.

## Experimental procedures

### Cultures

We used the Russian strain of the monogonont rotifer *B. manjavacas*, which was originally isolated from the Azov Sea, Russia and has been in culture for more than 30 years. Their diet consisted of the chlorophyte *Tetraselmis suecica,* which we maintained in 2-L flasks of bubbled 15 ppt artificial seawater f/2 medium (Guillard, 1975). We cultured rotifers and algae at 21 °C on a 12:12 h light/dark cycle. Under these conditions and depending on feeding regimen, amictic *B*. *manjavacas* females hatched from amictic eggs have a prereproductive (neonate) stage of 1–2 days, reach maximum reproductive output at day 4–6, produce 22–32 eggs and live an average of 8–12 days (Gribble & Mark Welch, 2013).

### Life history assays

To generate mothers for measurements of maternal effects in amictic females born from amictic mothers and in males born of mictic mothers, we started a batch culture of *B. manjavacas* from approximately 100 rotifers fed AL, which allows rotifers to grow to a concentration sufficient to produce a mixture of mictic and amictic females. After 1 week, we separated eggs from females by vortexing and moved the eggs into culture dishes containing AL food. After 5 h, we isolated hatched female neonates (the experimental mothers-to-be) individually into 1 mL of AL *T. suecica* culture in 24-well plates for the first 24 h, then transferred them to treatment food concentrations of 100% AL (6 × 10^5^ cells mL^−1^), 10% AL (6 × 10^4^ cells mL^−1^) or intermittent fasting (IF; alternating days of 100% AL food and 15 ppt Instant Ocean). To maintain cultures of *T. suecica* in semi-continuous log-phase growth during CR experiments, we replaced approximately 10% of the culture with f/2 medium daily (Guillard, 1975).

We conducted life table experiments as previously described (Gribble & Mark Welch, 2013). Every 24 h, we recorded survival, reproductive status (whether carrying eggs), and the number of offspring for each individual, who we then transferred to a new well with fresh algae of the appropriate concentration. At maternal ages of 3, 5 and 7 days, we isolated one female neonate (amictic experiment) or two male offspring (mictic experiment) hatched within the previous 24 h from each maternal female. All female offspring fed AL and had life table data collected as described above. We maintained male offspring in 15 ppt Instant Ocean without *T. suecica*, as males do not eat, and a prior experiment had shown no significant difference between life span in 100% *T. suecica* with daily transfer (5.6 ± 1.4 days) or Instant Ocean without daily transfer (5.2 ± 1.4 days). Because of their shorter life span, we recorded survival of male offspring every 12 h.

### Body size measurement

To ascertain the effect of maternal age and CR on offspring body size, we measured offspring hatched at from mothers at the age of 3, 5 and 7 days. Growth of females of other *Brachionus* spp. approximately follows the Bertalanffy function, reaching 85–90% of maximum width and length between the first and second day of life (Carmona *et al*., 1989). Therefore, we collected female offspring at 24- and 48-h posthatching. As males do not change considerably in size after hatching, we collected male offspring at 24 h. We preserved offspring with Lugol’s solution, photographed individuals at 200× on a Zeiss Axioskop using an Axiocam and measured body length and width using calibrated Zeiss AxioVision 4.8.2 software. Body volume was approximated using a geometric conversion to a cylinder.

### Data analysis

Censored data were included in survivorship curves and daily reproduction curves. The primary reason for censoring individuals was loss during daily transfer. We determined significant differences between Kaplan–Meier survival curves using Mantel–Cox log-rank tests in Prism 6.0 and used Kruskal–Wallis with Dunn’s multiple comparison tests to test for significant differences in mean life span, mean fecundity and mean volume.

## Supporting Information

**Table S1** Life span, fecundity and body size of mothers and offspring.
