# Supplementary material for: Maternal caloric restriction partially rescues the deleterious effects of advanced maternal age on offspring
Source: Aging Cell. 2014 Mar 24;13(4):623–30. doi: 10.1111/acel.12217 (PMC4116445; doi:10.1111/acel.12217)
Supplement: Supplementary file 1 — Table S1 Life span, fecundity and body size of mothers and offspring. [file acel0013-0623-sd1.docx]

| **Cohort** | **Maternal CR Regimen** | **Mean Lifespan (d)** | **Median Lifespan (d)** | **Maximum Lifespan**  **(95^th^ percentile)** | **Mean Fecundity**  **(n offspring ind^-1^)** | **n (Lifespan and Fecundity)** | **24 h Mean Volume (x 10^5^ µm^3^)** | **48 h Mean Volume (x 10^5^ µm^3^)** |
| --- | --- | --- | --- | --- | --- | --- | --- | --- |
| Amictic Mothers | 100% | 9.1 (0.36) | 9 | 16.0 | 29.7 (0.58) | 35 | n/a | n/a |
| Amictic Mothers | 10% | **10.1 (0.29)** | 10 | 15.0 | 30.5 (0.90) | 38 | n/a | n/a |
| Amictic Mothers | IF | **11.6 (0.51)** | 11 | 22.0 | 28.1 (1.1) | 39 | n/a | n/a |
| D3 Females | 100% | 10.9 (0.46) | 10 | 16.9 | 30.0 (0.90) | 41 | 67.5 (3.2) | 98.6 (2.5) |
| D3 Females | 10% | 10.3 (0.52) | 10 | 16.1 | **27.4 (0.56)** | 38 | 61.9 (3.1) | 107.3 (2.4) |
| D3 Females | IF | 11.2 (0.42) | 12 | 14.0 | 28.2 (0.74)) | 17 | 64.8 (3.6) | 98.7 (2.9) |
| D5 Females | 100% | 7.9 (0.36) | 7 | 13.7 | 22.3 (0.93) | 42 | 133.0 (6.0) | 157.9 (6.1) |
| D5 Females | 10% | **9.2 (0.31)** | 9 | 14.4 | **26.2 (0.51)** | 43 | 153.5 (4.7) | 173.7 (3.9) |
| D5 Females | IF | **9.3 (0.42)** | 9 | 14.9 | **25.6 (0.94)** | 40 | **99.6 (5.9)** | **129.4 (5.2)** |
| D7 Females | 100% | 7.1 (0.25) | 7 | 9.9 | 16.3 (0.82) | 41 | 148.3 (7.4) | 214.4 (5.4) |
| D7 Females | 10% | **8.3 (0.35)** | 8 | 12.0 | **19.9 (1.2)** | 39 | 156.8 (8.7) | 219.4 (6.4) |
| D7 Females | IF | **8.2 (0.36)** | 8 | 13.0 | **19.9 (1.1)** | 42 | 140.3 (7.1) | **152.4 (7.0)** |
| Mictic Mothers | 100% | 8 (0.32) | 9 | 10.8 | 25.6 (1.1) | 43 | n/a | n/a |
| Mictic Mothers | 10% | **9.6 (0.39)** | 9 | 14.0 | **28.8 (0.78)** | 44 | n/a | n/a |
| Mictic Mothers | IF | **14.6 (0.87)** | 14 | 24.1 | **14.6 (0.87)** | 37 | n/a | n/a |
| D3 Males | 100% | 5.6 (0.14) | 5 | 8.0 | n/a | 88 | 6.8 (0.14) | n/a |
| D3 Males | 10% | 5.5 (0.15) | 5 | 8.0 | n/a | 88 | 6.8 (0.14) | n/a |
| D3 Males | IF | 5.3 (0.14) | 5 | 7.5 | n/a | 70 | 6.8 (0.21) | n/a |
| D5 Males | 100% | 5.3 (0.18) | 5 | 8.0 | n/a | 84 | 8.9 (0.22) | n/a |
| D5 Males | 10% | 5.3 (0.18) | 5.5 | 8.0 | n/a | 85 | 8.2 (0.17) | n/a |
| D5 Males | IF | 5.2 (0.19) | 5.5 | 7.5 | n/a | 75 | **7.5 (0.18)** | n/a |
| D7 Males | 100% | 5.3 (0.24) | 6 | 8.0 | n/a | 49 | 10.8 (0.30) | n/a |
| D7 Males | 10% | 4.9 (0.20) | 4.5 | 8.0 | n/a | 70 | **9.8 (0.36)** | n/a |
| D7 Males | IF | 4.8 (0.20) | 4 | 7.5 | n/a | 70 | n/a | n/a |
